# Construction and validation of a scenario for recognizing sepsis by nursing students: a methodological study

**DOI:** 10.1590/0034-7167-2022-0537

**Published:** 2023-10-09

**Authors:** Jane Walkíria da Silva Nogueira, Marcia Cristina da Silva Magro

**Affiliations:** I Universidade de Brasília. Brasília, Distrito Federal, Brazil

**Keywords:** Validation Study, Sepsis, Simulation Training, Nursing Education, Nursing Students, Estudio de Validación, Sepsis, Entrenamiento Simulado, Educación en Enfermería, Estudiantes de Enfermería, Estudo de Validação, Sepse, Treinamento por Simulação, Educação em Enfermagem, Estudantes de Enfermagem.

## Abstract

**Objective:**

To build and validate a clinical simulation scenario for teaching Nursing students about early recognition of signs and symptoms of sepsis in the context of the emergency unit.

**Methods:**

Methodological study developed in two phases: construction of a simulated scenario and content validation by expert judges. For data analysis, the Content Validity Index (CVI) was calculated considering agreement equal to or greater than 80%. The minimum acceptable CVI value for scenario validation was 1.0.

**Results:**

The simulation scenario proved to be appropriate, with a global Content Validity Index equal to 1. Some adjustments related to the clarity of the wording were necessary, as suggested by the judges**. Conclusions:** A medium-complexity, high-fidelity scenario was successfully constructed and validated for teaching early recognition of sepsis signs and symptoms.

## RESUMO

**Objetivo:**

Construir e validar um cenário de simulação clínica para o ensino de estudantes de Enfermagem sobre reconhecimento precoce de sinais e sintomas de sepse no contexto da unidade de emergência.

**Métodos:**

Estudo metodológico desenvolvido em duas fases: construção de cenário simulado e validação do conteúdo por juízes especialistas. Para análise dos dados, foi calculado Índice de Validade de Conteúdo (IVC) considerando concordância igual ou superior a 80%. O valor mínimo aceitável de IVC para validação do cenário foi de 1,0.

**Resultados:**

O cenário de simulação mostrou se apropriado, com Índice de Validade de Conteúdo global igual a 1. Alguns ajustes relacionados à clareza da redação foram necessários, conforme sugestão dos juízes**. Conclusões:** Foi construído e validado com êxito um cenário de média complexidade e alta fidelidade para o ensino sobre o reconhecimento precoce de sinais e sintomas de sepse.

## RESUMEN

**Objetivo:**

Construir y validar un escenario de simulador clínico para la enseñanza de estudiantes de Enfermería sobre reconocimiento precoz de signos y síntomas de sepsis en el contexto de la unidad de urgencia.

**Métodos:**

Estudio metodológico desarrollado en dos fases: construcción de escenario simulado y validez de contenido por jueces especialistas. Para análisis de los datos, fue calculado Índice de Validez de Contenido (IVC) considerando concordancia igual o superior a 80%. El valor mínimo aceptable de IVC para validez del escenario fue de 1,0.

**Resultados:**

El escenario de simulado se mostró apropiado, con Índice de Validez de Contenido global igual a 1. Algunos ayustes relacionados a la claridad de la redacción fueron necesarios, conforme sugestión de los jueces.

**Conclusiones:**

Fue construido y validado con éxito un escenario de mediana complejidad y alta fidelidad para la enseñanza sobre el reconocimiento precoz de signos y síntomas de sepsis.

## INTRODUCTION

Sepsis is considered a public health problem in Brazil and in the world, constituting a challenge to be faced by public policies. It is currently defined as a life-threatening organ dysfunction caused by a dysregulated host response to infection^(1)^.

The multicentric study Spread, conducted by ILAS (Latin American Institute of Sepse), showed that one third of beds in intensive care units are occupied by patients with sepsis and septic shock, in which the overall lethality was 55%^(2)^.

Between 47 and 50 million people are affected annually by this syndrome. Estimated costs associated with the treatment are between US$26,000 and US$32,000 in the United States and US$9,600 per patient in Brazil^(3)^.

Several reasons may be associated with the high mortality rate due to sepsis in Brazil, highlighting the possible unpreparedness of health professionals to quickly and correctly identify cases of sepsis or septic shock and the consequent delay in starting treatment^(3-4)^.

In this perspective, national^(5-6)^ and international^(7-8)^ studies have shown a deficit in knowledge about the recognition, development and management of sepsis by nursing students and nurses.

However, the adoption of active and immersive educational strategies for learning about sepsis can favor the improvement of knowledge on this topic, provide early identification of its signs and symptoms, as well as the differentiation of evolutionary phases by nursing, improving the patient prognosis^(9)^.

In this regard, teaching based on clinical simulation, through repetitive practice (repetition until excellence) and experiencing clinical situations in a controlled environment, proves to be crucial for the training and formation of safer and more capable professionals, for example, identify the signs and symptoms of sepsis. Simulation as an educational strategy stimulates reflection, critical thinking and the ability to make clinical decisions, as well as the development of psychomotor skills in Nursing students^(10)^.

The construction of well-planned and systematized scenarios represents an alternative that favors the approximation of nursing students to the reality of clinical practice. In addition, the validation process through the consolidation of reliability by expert judges can strengthen educational strategies and improve the relationship between theory and practice, aiming to contribute to the formation of more reflective and critical nurses, with skills for the early recognition of sepsis.

## OBJECTIVE

Build and validate a clinical simulation scenario of medium complexity and high fidelity for teaching Nursing students about early recognition of signs and symptoms of sepsis in the context of the emergency unit.

## METHODS

### Ethical aspects

The study was developed in compliance with national and international standards of ethics in research with human beings, in accordance with Resolution 466/12, after authorization by the Research Ethics Committees of Faculdade Ciências da Saúde and Faculdade de Ceilândia, both at the University of Brasilia.

### Study design, period, and place

Methodological study, with cross-sectional design and quantitative approach, following the recommendations of the Simulation - Based Research Extensions for the Strengthening the Reporting of Observational Studies Epidemiology (STROBE) and the International Nursing Association for Clinical Simulation and Learning (INACSL). The study was carried out from August to November 2021 at a public university in the Federal District and followed the following steps: construction of the clinical simulation scenario; content validation by expert judges.

### Population, inclusion, and exclusion criteria

The selection of expert judges took place through intentional non-probabilistic sampling. Inclusion criteria were based on curriculum analysis according to Fehring’s framework^(11)^. For selection in the Lattes curriculum, the following search criteria were adopted: being a nurse, degree level (specialist, master, or doctor), clinical care practice in the area of nursing care in critical and risk situations, clinical simulation and nursing teaching.

The minimum score considered valid was 7 points(11), out of a total of 14 points distributed in the items: doctoral degree in Nursing or related areas = 4 points; master’s degree in Nursing or related areas = 4 points; specialization in Nursing in a general and/or cardiology adult intensive care unit = 2 points; clinical care practice or teaching in the area of critical care = 2 points; clinical simulation experience = 1 point; knowledge about sepsis = 1 point.

After consulting the Lattes curriculum, 13 judges were invited by email, with acceptance of ten by signing the Free and Informed Consent Form. According to Pasquali^(12)^, a minimum number of six specialists is required. After acceptance, the judge’s characterization questionnaire was sent (name, gender, age, professional training, time since graduation and professional practice; area of expertise, title, experience with clinical simulation and with the topic of sepsis), script of the clinical scenario simulated and content validation form for experts. The deadline for returning the instruments by the specialists was 15 days.

### Study protocol

The first step in the textual construction of the scenario was the choice of the theme sepsis, defined as the presence of at least two signs of systemic inflammatory response syndrome (SIRS) and/or an organ dysfunction^(1)^.

Next, the textual content was elaborated according to the following aspects: prior knowledge of the learner; learning objectives; theoretical foundation of the activity; scenario preparation and development; debriefing; and evaluation^(13)^.

The learner’s prior knowledge for experiencing the simulated scenario was defined by the pre-existing cognitive structure based on curricular content^(13)^ and access to didactic material in the form of a booklet on the theme of sepsis.

The learning objectives were based on the need for early recognition of signs and symptoms of sepsis using the SIRS and organ dysfunction criteria. The theoretical foundation of the simulated clinical scenario was guided by the best levels of evidence proposed by the guidelines of the Surviving Sepsis Campaign - SSC (Surviving Sepsis Campaign 2021)^(1)^.

The stage of preparing the scenario of a patient with suspected sepsis sought to emphasize the main signs and symptoms, such as abdominal pain, fever, tachypnea, and disjointed words.

The environment reproduced was an emergency room with a hospital-type bed, medical equipment/materials (cardiopulmonary arrest cart, defibrillator, multiparameter monitor, gas ruler, pulse oximeter, non-invasive blood pressure device, stethoscope, thermometer, among others) to offer realism to the scene. The patient was mimicked by a high-fidelity mannequin (traditional Sim Man^®^) capable of physiological responses. The estimated time for the debriefing was 30 minutes. The structured model was chosen (emotional, descriptive, evaluative, analytical, conclusive stage)^(14)^, as it allows reflection on the simulated experience.

The traditional Delphi technique was used to validate the content of the research instruments by expert judges^(15)^. Two rounds were necessary, the second being for evaluating the reformulated version after the contributions made by the experts in the first round.

The judges evaluated each instrument item (simulated scenario) according to the following criteria: a) clarity: question containing important information for achieving the study objectives, stated in an understandable manner; b) scope: question that incorporates or includes information relevant to the achievement of the research objective; c) organization: arrangement of questions and alternatives, as well as their content; d) pertinence: relevant question to achieve the objective of the research. These criteria were analyzed considering a Likert-type scale, with four response levels: Irrelevant = 1; Little relevant = 2; Quite relevant = 3; Extremely relevant = 4. There was also an open field for comments, should the judge deem it necessary.

### Analysis of results and statistics

The results were analyzed using the R Core Team 2021 software (Version 4.1.0). For analysis regarding the degree of agreement of the items, the Content Validity Index was calculated and the Exact Binomial test was applied to each item in each criterion (organization, clarity, scope and pertinence), verifying the proportion of agreement between the judges, adopted as equal to or greater than 80% (P ≥ 80%)^(16)^ and significance (α) of 5%. Thus, p values greater than 0.05 indicate that there was agreement among the judges on the items in each criterion. In addition, the Mean Scale Content Validity Index (S-CVI-AVE) and the Percentage of Items with Unanimous Agreement (S-CVI-UA) were calculated. The minimum acceptable CVI value for scenario validation was 1.0, following the literature recommendation for a body of six judges or less^(16)^.

## RESULTS

The scenario content validation process was carried out by a total of ten expert nurse judges with a mean age of 36.2±8.3 years. Of these, 80% were women, with an average of 13.1±8.0 years since graduation and 12.5±8.4 years of professional practice. Of the total, 50% worked in a general intensive care unit and 40% declared themselves professors of higher education in Nursing. All (100%) reported clinical experience with sepsis. Regarding clinical simulation, 90% of participants reported experience with simulated teaching. The predominant academic title was doctors (50%), while 40% of the judges declared themselves specialists (10% in cardiology and 30% in general intensive care unit), and 10% were masters.

Two rounds were carried out for the evaluation of the expert judges in order to obtain an agreement of at least 80%. In the first round, there were 14 items in the instrument related to the simulated clinical scenario. In all criteria and in all items, the CVI ranged from 90% to 100%, with p > 0.05 indicating agreement greater than or equal to 80% for item permanence. Regarding the validity of the scale, the mean S-CVI ranged from 97% to 98% between the criteria, and the percentage of unanimity varied between 71% and 79% between the criteria.

In the second round, the 14 items of the instrument related to the simulated clinical scenario remained. Regarding the Organization, Clarity, and Comprehensiveness criteria, the CVI was 100% with p > 0.05 in all items, indicating agreement greater than or equal to 80% for the permanence of the item. In the Pertinence criterion, the CVI ranged from 89% to 100%, with p > 0.05 in all items, indicating agreement greater than or equal to 80% for the permanence of the item. Regarding the validity of the scale, both the mean S-CVI ranged from 99% to 100% and the percentage of unanimity ranged from 93% to 100% between the criteria (Table 1). After evaluating the judges, all items of the simulated clinical scenario were considered validated.

**Table 1:** Judges’ assessment of the organization, clarity, appearance, and relevance of the simulation scenario, the CVI and the p-value of each item, Brasília, Distrito Federal, Brazil, 2021

Evaluated items	Delphi Technique First Assessment (first round)								Delphi Technique Second Assessment (second round)							
	Organization		Clarity		Appearance		Relevance		Organization		Clarity		Appearance		Relevance	
	CVI^*^	*p*	CVI^*^	*p*	CVI^*^	*p*	CVI^*^	*p*	CVI^*^	*p*	CVI^*^	*p*	CVI^*^	*p*	CVI^*^	*p*
1. Scenario title	100	1.000	100	1.000	100	1.000	100	1.000	100	1.000	100	1.000	100	1.000	100	1.000
2. Public	100	1.000	100	1.000	100	1.000	100	1.000	100	1.000	100	1.000	100	1.000	100	1.000
3. Prior experience of the learner	100	1.000	100	1.000	100	1.000	100	1.000	100	1.000	100	1.000	100	1.000	100	1.000
4. Learning objectives	90	0.893	100	1.000	100	1.000	100	1.000	100	1.000	100	1.000	100	1.000	89	0.866
5. Simulation time	100	1.000	100	1.000	100	1.000	100	1.000	100	1.000	100	1.000	100	1.000	100	1.000
6. Human Resources	100	1.000	100	1.000	100	1.000	100	1.000	100	1.000	100	1.000	100	1.000	100	1.000
7. Material resources/equipment	100	1.000	100	1.000	100	1.000	100	1.000	100	1.000	100	1.000	100	1.000	100	1.000
8. Documentation	90	0.893	90	0.893	90	0.893	90	0.893	100	1.000	100	1.000	100	1.000	100	1.000
9. Scenario preparation	90	0.893	90	0.893	90	0.893	90	0.893	100	1.000	100	1.000	100	1.000	100	1.000
10. Briefing	100	1.000	100	1.000	100	1.000	100	1.000	100	1.000	100	1.000	100	1.000	100	1.000
11. Scenario development: description of the clinical case	100	1.000	100	1.000	100	1.000	100	1.000	100	1.000	100	1.000	100	1.000	100	1.000
12. Scenario development: information in the medical record	100	1.000	100	1.000	100	1.000	100	1.000	100	1.000	100	1.000	100	1.000	100	1.000
13. Scene schedule	90	0.893	90	0.893	90	0.893	90	0.893	100	1.000	100	1.000	100	1.000	100	1.000
14. Debriefing	100	1.000	100	1.000	100	1.000	100	1.000	100	1.000	100	1.000	100	1.000	100	1.000
S-CVI-AVE^**^	97		98		98		98		100		100		100		99	
S-CVI-UA^***^	71		79		79		79		100		100		100		93	

* CVI - Content Validity Index, Exact Binomial Test.

** S-CVI-AVE - Mean Scale Content Validity Index.

*** S-CVI-UA - Percentage of Item with Unanimous Agreement.

Regarding the qualitative analysis of the judges in relation to the scenario of the simulation session, two (20%) suggested reorganizing the text regarding the learning objectives, to provide greater clarity. Only one (10%) suggested, in the secondary objectives, removing the item related to the principles of biosafety, measurement and evaluation of vital signs, as these are considered the student’s previous skill.

As for the materials/equipment used in the scenario, the inclusion of some items was suggested, such as: the gas ruler (1; 10%); blood culture bottle for fungi and kit for indwelling bladder catheterization (2; 20%); clipboard, screen, infectious waste, hamper and emergency trolley with seal (1; 10%); lactated Ringer’s solutions and 5% glucose solution (1; 10%); heparinized syringe for arterial blood gases (1; 10%).

In the item referring to the documentation used in the simulated scene, there was a suggestion of adding the nursing record sheet by only one (10%) of the judges. In the detailed description of the scenario, one of the ten judges suggested the inclusion of statements by participants in the simulated activity. In this sense, a script was prepared with dialogue from the actors/collaborators participating in the simulated scenario. Below, Chart 1 represents a summary of the final version of the validated simulated scenario on sepsis with the contributions of the expert judges.

**Chart 1:** Brief description of the final version of the validated scenario “Nurses’ role in early recognition of signs and symptoms of sepsis”, Brasília, Federal District, Brazil, 2022

Scenario title	Nurses’ role in the early recognition of signs and symptoms of sepsis
**Public**	Nursing Students
**Prior knowledge of the learner**	Skills in caring for patients with suspected sepsis
**Learning objectives**	**Primary:** recognize the risk of sepsis and make decisions. **Secondary**: collect data in order to identify the patient with suspected sepsis; communicate effectively with the patient and their family members in search of signs and symptoms suggestive of sepsis; when suspecting sepsis, open a protocol and call the medical team; perform and prioritize nursing care.
**Simulation time**	***Briefing:*** 5 minutes; ***Scenario***: 15 minutes; ***Debriefing*:** 30 minutes
**Human Resources:**	Three monitors to assume the following roles: simulated patient (high-fidelity simulator control room); doctor who will appear when requested by the nurse; patient’s child; two teaching facilitators with experience or training in simulation.
Material resources/equipment	Hospital bed with side rails, bedding, pillow, gas ruler, auxiliary table, two-step ladder, hospital gown, parrot, serum holder, infectious waste, sealed emergency trolley, procedure and sterile gloves, antiseptics, equipment personal protective equipment, gauze, stainless steel tray, saline, glucose and ringer lactate solutions, needles (40×12; 30×7; 13×45 mm), heparinized syringe, syringes (3, 5, 10, 20 mL), equipment of serum and two-way connector, label for serum identification, hypoallergenic microporous tape, tourniquet, flexible catheter for venipuncture (#20, 22), transparent film, sachet of 70% alcohol, antibiotics and analgesics, flasks for blood culture, tubes for blood collection, kit for indwelling urinary catheter, electrodes, multiparametric monitor, stethoscope, sphygmomanometer, pulse oximeter, spectacle-type nasal oxygen catheter, oxygen humidifier, distilled water, discharge patient simulator dummy fidelity.
**Documentation**	Medical prescription, nursing record, sheet with laboratory results: blood glucose: 90 mg/dL (RV: 65 to 99 mg/dL); leukocytes: 25,100/cell.mm3 (RV: 5,000-10,000 mm3); lactate: 2.5 mmol/L (RV: 0.3-2.4 mmol/L); imaging exam with report (abdominal tomography): moderate-volume ascites and densification of the peritoneal adipose planes (inflammatory process); splenomegaly.
**Scenario preparation**	
**Theme**: Sepsis with abdominal focus. **Scenario fidelity**: High fidelity. **Characterization of actors/collaborators:** Laerdal SimMan^®^ Traditional high-fidelity patient simulator, featuring hospital-appropriate attire (gown with open back); patient’s son (monitor wearing jeans and a short-sleeved shirt); doctor (monitor wearing jeans, short-sleeved shirt, and white coat). **Physical space**: The scene takes place in an environment characterized as the box bed of the emergency unit. **Scenario complexity:** Medium complexity. **Expected actions**: confirmation of the clinical case for sepsis, opening of the protocol and start of the first hour package. **Previous skills**: biosafety principles, measurement and analysis of vital signs, physical examination, communication and interaction with the patient, analysis of data provided by the patient, family, exams, medical records.	
**Scenario development:**	
**Clinical case description**	**JRS**, male, 65 years old, married, with a history of type 2 diabetes mellitus controlled with diet and regular physical activity. He was admitted to the emergency room accompanied by his son and previous history of hospitalization for laparoscopic cholecystectomy with hospital discharge two days ago. One day ago, oliguria and abdominal pain with the use of analgesics (paracetamol) at home. According to the son, today the patient woke up with a fever of 38^o^C and worsening abdominal pain, tachypnea and talking about strange things.
**Medical record information**	**Clinical History:** Type 2 diabetes mellitus, laparoscopic cholecystectomy. Denies allergies. **Medications in use:** Paracetamol every 6 hours in the last 24 hours. **Anthropometric data** ; Body mass: 85kg
**Devices attached to the simulator**	Hospital gown, identification wristband, orange color risk rating wristband
***Debriefing***	Structured
**Assessment**	Students’ practical performance

## DISCUSSION

The validation of educational materials has gained relevance and attracted greater interest for the construction of the teaching-learning process and qualified training for the job market, with a potential reduction in academic evasion^(17-18)^.

Simulation-based approaches encourage the active involvement of students in building knowledge and developing their skills in various contexts^(19)^. From this perspective, a scenario was constructed and validated regarding the admission of a patient with signs and symptoms of sepsis in the context of an emergency unit, based on a guiding script^(13)^ and based on the guidelines of the International Nursing Association for Clinical Simulation and Learning^(20)^.

Content validation was carried out by a group of specialists with qualification/education in the areas of clinical simulation and sepsis, using the Delphi technique^(15)^, which enabled the consensus of specialists, in addition to the analysis of agreement of the items that reproduce with confidence the recognition of signs and symptoms of sepsis by nurses.

The Delphi technique has advantages such as the possibility of accessing geographically distant people, the low operational cost, the possibility of interaction between researcher and participants, the sharing of opinions and ideas, and the production of an instrument with high quality and specificity. On the other hand, it is susceptible to some disadvantages such as the delay in returning the questionnaires, difficulties in the composition of experts and the need for several rounds to establish a final consensus^(15)^.

The validation results were positive, and the experts’ suggestions added greater quality to the scenario, which strengthened realism and expanded the specific information related to the theme. Evidence on sepsis screening and care in developing countries is insufficient to inform implementation practices in healthcare settings^(21)^. So, expanding knowledge and professional qualification through active and immersive methodologies, such as simulation, represents an alternative for the systematization of clinical practices, mainly in emergency and intensive care units..

The number of judges included in the study favored the achievement of the Content Validity Index (CVI) at a value of 1, as recommended in the literature^(16)^, which represents a level of agreement between expert judges greater than 80% of the items evaluated in the questionnaire referring to the simulated scenario.

The clarity of the scenario’s learning objectives showed improvement, given the progression obtained in the mean CVI from the first round (98%) to the second round (100%), which reveals consensual agreement among specialists and availability for use. Certainly, the objectives must be specific, measurable, achievable, realistic and achievable in a timely manner^(22)^, as shown in the results. In this context, it becomes possible to improve the chances of disseminating information and encouraging reflection^(23)^ regarding the systematization of care for sepsis using active, integrated and immersive strategies, such as simulation.

In addition, in scenario validation, the objectives in the first stage should guide the actions developed in the subsequent stages, which reinforces the importance of reaching the highest possible level of agreement. The validation method aims to assess whether the simulated scenario fulfills its purpose and whether it is reproducible as an innovative teaching tool^(24)^.

In the simulation session, the briefing represents the first pre-simulation stage and aims to provide information that guides and directs the participants. It is recognized that the presentation of the scenario and its possibilities can facilitate the understanding of the learning objectives in the execution and safety^(20,22)^.

Human resources must be provided for the scenario to be developed according to the learning objectives. Surely, simulation facilitators and simulation can contribute to the optimization of work structures and processes^(25)^. In some laboratories and/or simulation centers, mainly in the Brazilian context, it is still common to come across professors who do not have training in clinical simulation; however, participation and specialized technical support are essential to control and program the simulators and/or support the educator in methodological issues of structuring, setting up and executing simulated scenarios^(26)^.

Regarding material resources/equipment, adjustments were necessary to meet the suggestions of expert judges, such as the inclusion of materials and devices. The importance of educational and innovative technologies to improve the workforce is known, so the availability of equipment can optimize work systems^(25)^. In addition, a script was introduced with dialogue between the actors/collaborators to guide and ensure the quality of the dialogue between the participants and the patient simulator during the development of the simulated scene.

The complexity of the scenario, although moderate, was executed with realism, given the high-fidelity configuration to encourage better development of clinical reasoning and decision-making in the face of each sign and symptom presented by the patient. For a better use of the strategy, it is necessary to consider the level of previous knowledge of the participants, which must be compatible with the complexity of the scenario. Clinical simulation is seen as a didactic-pedagogical support technique that provides curriculum integration and associates prior knowledge with practical experience^(27)^.

Still in relation to the development of the scenario, it is important that the facilitator plans the skills and abilities that must be improved or developed by the participant of the simulated activity, considering previous knowledge and experience.

The use of structured debriefing with “good judgment” contributed to giving students the opportunity to express themselves actively, with consequent appreciation of their point of view. At this stage of the simulation session, it is possible to treat the mistakes made as a learning opportunity, which makes it possible to improve critical and constructive judgment and favor reflective thinking^(28)^.

Modern pedagogy has revealed the importance of learning through pedagogical innovations, including considering the technological engagement of students in the 21st century. So, exploring tools and developing potentials in learning contexts to improve knowledge has been increasing in the area of health sciences^(19)^.

Therefore, the construction and validation of a scenario involving the topic of sepsis can be a support tool and additional support for positive learning outcomes through situations/cases that provide greater student development and the structuring of knowledge and skills that give rise to reflection in future nurses. This is because such learning techniques aim to teach these professionals to identify patients at risk of sepsis, contribute significantly to minimizing the risk of delay in diagnosis and initiate conduct in a targeted manner.

### Study limitations

Limitations were related to restrictions on access to spaces for validation and testing of the scenario, imposed by the covid-19 pandemic; and the difficulty of compliance with the response time by the judges.

### Contributions to the area of nursing, health, or public policy

The scenario built and validated in this study can be used as a facilitating educational tool by professors from higher education institutions in Nursing or in training programs for nurses working in emergency or intensive care units. The development of cognitive, affective, and psychomotor skills in emergency situations such as sepsis is essential, considering that the absence of critical thinking, problem solving and decision-making ability can seriously harm patients. So, qualifying future nurses for the early identification of sepsis gains prominence for individualized, qualified, and safe care.

## CONCLUSION

The clinical simulation scenario of medium complexity and high fidelity for teaching Nursing students about the role of nurses in the early recognition of signs and symptoms of sepsis was successfully constructed and validated. It was prepared based on protocols based on the best levels of evidence, being validated by expert judges with clinical care practice in the area of nursing care in critical and risk situations, clinical simulation and nursing teaching. The number of judges included in the study favored reaching the Content Validity Index at a value of 1, which represents a level of agreement between judges greater than 80% of the items evaluated in the instrument referring to the simulated scenario.

## References

[1] Evans L, Rhodes A, Alhazzani W, Antonelli M, Coopersmith CM, French C (2021). Surviving sepsis campaign: international guidelines for management of sepsis and septic shock 2021. Crit Care Med.

[2] Machado FR, Cavalcanti AB, Bozza FA, Ferreira EM, Angotti Carrara FS, Sousa JL (2017). The epidemiology of sepsis in Brazilian intensive care units (the sepsis prevalence assessment database, spread): an observational study. Lancet Infect Dis.

[3] Viana RAPP, Machado FR, Sousa JLA. (2020). Sepse um problema de saúde pública: atuação e colaboração da enfermagem na rápida identificação e tratamento da doença.

[4] Zonta FNS, Velasquez PGA, Velasquez LG, Demetrio LS, Miranda D, Silva MCBD. (2018). Características epidemiológicas e clínicas da sepse em um hospital público do Paraná. Rev Epidemiol Control Infect.

[5] Goulart LS, Ferreira MA, Sarti ECFB, Sousa ÁFL, Ferreira AM, Frota OP. (2019). Are nurses updated on the proper management of patients with sepsis?. Esc Anna Nery.

[6] Silva DF, Brasil MHF, Santos GCV, Guimarães KSL, Oliveira FMRL, Leal NPR (2021). Conhecimento dos enfermeiros emergencistas acerca do protocolo clínico de sepse. Rev Enferm UFPE.

[7] Harley A, Johnston ANB, Denny KJ, Keijzers G, Crilly J, Massey D. (2019). Emergency nurses’ knowledge and understanding of their role in recognising and responding to patients with sepsis: a qualitative study. Int Emerg Nurs.

[8] Harley A, Massey D, Ullman AJ, Reid-Searl K, Schlapbach LJ, Takashima M (2021). Final year nursing student’s exposure to education and knowledge about sepsis: A multi-university study. Nurs Educ Today.

[9] Oliveira SC, Corrêa BT, Dodde HN, Pereira GL, Aguiar BGC. (2019). The nurse approach towards the detection of antecedent signs and symptoms of sepsis in patients at a nursing ward. Rev Pesqui Cuid Fundam.

[10] Carvalho LR, Zem-Mascarenhas SH. (2020). Construção e validação de um cenário de simulação sobre sepse: estudo metodológico. Rev Esc Enferm USP.

[11] Fehring RJ. (1987). Methods to validate nursing diagnoses. Heart Lung.

[12] Medeiros RKS, Ferreira MA, Pinto DPSR, Vitor AF, Santos VEP, Barichello E. (2015). Modelo de validação de conteúdo de Pasquali nas pesquisas em Enfermagem. Rev Enferm Ref.

[13] Fabri RP, Mazzo A, Martins JCA, Fonseca AS, Pedersoli CE, Miranda FBG (2017). Development of a theoretical-practical script for clinical simulation. Rev Esc Enferm USP.

[14] Nascimento JSG, Oliveira JLG, Alves MG, Braga FTMM, Góes FSN, Dalri MCB. (2020). Debriefing methods and techniques used in nursing simulation. Rev Gaúcha Enferm.

[15] Williams PL, Webb C. (1994). The Delphi technique: a methodological discussion. J Adv Nurs.

[16] Alexandre NMC, Coluci MZO. (2011). Validade de conteúdo nos processos de construção e adaptação de instrumentos de medidas. Ciênc Saúde Coletiva.

[17] Bublitz S, Guido LA, Kirchhof RS, Neves ET, Lopes LFD. (2015). Sociodemographic and academic profile of nursing students from four brazilian institutions. Rev Gaúcha Enferm.

[18] Diesel A, Baldez ALS, Martins SN. (2017). Os princípios das metodologias ativas de ensino: uma abordagem teórica. Rev Thema.

[19] Regmi K, Jones L. (2020). A systematic review of the factors - enablers and barriers - affecting e-learning in health sciences education. BMC Med Educ.

[20] (2016). Inacsl standards of best practice: simulations outcomes and objectives. Clin Simul Nurs.

[21] Alberto L, Marshall AP, Walker RM, Pálizas F, Aitken LM. (2021). Improving sepsis screening and care in a developing nation health setting: a description of implementation. Nurs Health Sci.

[22] Costa RRO, Medeiros SM, Martins JCA, Coutinho VRD. (2019). Perceptions of nursing students on the structural dimensions of clinical simulation. Sci Med.

[23] Pissinati PSC, Évora YDM, Marcon SS, Mathias TAF, Fonseca LF, Haddad MCFL. (2021). Content and usability validation of the Retire with Health web software. Rev Bras Enferm.

[24] Andrade PON, Oliveira SC, Morais SCRV, Guedes TG, Melo GP, Linhares FMP. (2019). Validation of a clinical simulation setting in the management of postpartum haemorrhage. Rev Bras Enferm.

[25] Dieckmann P, Torgeirsen K, Qvindesland SA, Thomas L, Bushell V, Ersdal HL. (2020). The use of simulation to prepare and improve responses to infectious disease outbreaks like COVID-19: practical tips and resources from Norway, Denmark, and the UK. Adv Simul.

[26] Carreiro BO, Romão LGB, Costa RO. (2021). Construction and validation of simulated basic life support scenarios in primary care. Mundo Saúde.

[27] Jeffries PR, National League for Nursing (2012). Simulation in nursing education: from conceptualization to evaluation.

[28] Bortolato-Major C, Mantovani MF, Felix JVC, Boostel R, Silva ÂTM, Caravaca-Morera JA. (2019). Debriefing evaluation in nursing clinical simulation: a cross-sectional study. Rev Bras Enferm.

